# Endogenous salicylic acid shows different correlation with baicalin and baicalein in the medicinal plant *Scutellaria baicalensis* Georgi subjected to stress and exogenous salicylic acid

**DOI:** 10.1371/journal.pone.0192114

**Published:** 2018-02-13

**Authors:** Hu Su, Shurui Song, Xin Yan, Limin Fang, Bin Zeng, Youlin Zhu

**Affiliations:** 1 Life Science Department, Nanchang University, Nanchang, Jiangxi Province, China; 2 Life Science Department, Jiangxi Science and Technology Normal University, Nanchang, Jiangxi Province, China; Estacion Experimental del Zaidin, SPAIN

## Abstract

Salicylic acid (SA) is synthesized via the phenylalanine lyase (PAL) and isochorismate synthase (ICS) pathways and can influence the stress response in plants by regulating certain secondary metabolites. However, the association between SA and particular secondary metabolites in the Chinese medicinal plant *Scutellaria baicalensis* Georgi is unclear. To elucidate the association between SA and the secondary metabolites baicalin and baicalein, which constitute the primary effective components of *S*. *baicalensis*, we subjected seedlings to drought and salt stress and exogenous SA treatment in a laboratory setting and tested the expression of *PAL* and *ICS*, as well as the content of free SA (FSA), total SA (TSA), baicalin, and baicalein. We also assessed the correlation of FSA and TSA with PAL and ICS, and with baicalin and baicalein accumulation, respectively. The results indicated that both FSA and TSA were positively correlated with PAL, ICS, and baicalin, but negatively correlated with baicalein. The findings of this study improve our understanding of the manner in which SA regulates secondary metabolites in *S*. *baicalensis*.

## Introduction

Plants are frequently challenged by a variety of stresses associated with drought, salt, and temperature. Salicylic acid (SA) can regulate the physiological processes of plants under a variety of stresses, thereby altering their resistance to these challenges [1, 2]. During this regulatory process, SA induces the expression of many defense genes [3, 4], which results in alterations in the accumulation of some secondary metabolites. One of the major functions of these secondary metabolites is to improve the tolerance of plants to stress [5]. The accumulation of several of these metabolites has been well studied in plants treated with SA in response to pathogen infection, and some have been functionally identified as antimicrobial compounds. For instance, SA treatment induced the synthesis of phenol-2,4-bis (1,1-dimethylethyl) in avocado roots, which strengthened their defense against the soil-borne water mold *Phytophthora cinnamomi* [6]. SA is involved in camalexin synthesis in *Arabidopsis thaliana* leaves upon *Pseudomonas syringae* infection [7], and has also been found to regulate certain secondary metabolites associated with the abiotic stress response (e.g., ozone, salt, UV, or heat). For example, SA mitigated salinity stress effects by increasing the total phenolic, chlorophyll, carbohydrates, and proline contents of *Rosmarinus officinallis* L. leaves along with decline in sodium and chloride [8]. SA counteracts the effects of UV stress on *Capsicum annuum* by increasing rutin production [9]. It also reduces malondialdehyde accumulation and alleviates cadmium stress in perennial ryegrass [10]. Under high temperature stress, *Populus* transformed with the *FD-irp9* gene, which results in the accumulation of high levels of SA, accumulated phenylpropanoids and phenolic glycosides and exhibited metabolic patterns that resembled those of high temperature-treated wild-type plants [11].

Many studies have assessed the function of SA as a key signaling molecule in response to both abiotic and biotic stress factors. SA, which is synthesized via the phenylalanine lyase (PAL) and isochorismate synthase (ICS) pathways [12], controls resistance by regulating the accumulation of some secondary metabolites in plants.

**Fig 1:**
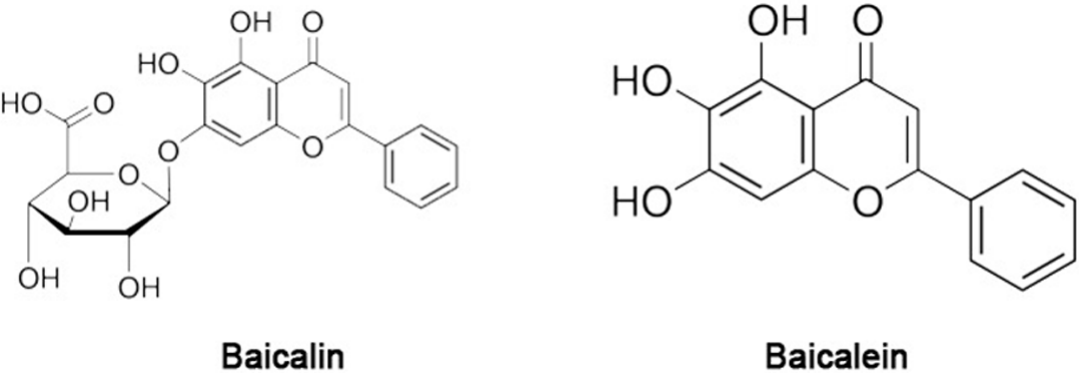
Structure of baicalin and baicalein.

However, the association between SA and the secondary metabolites synthesized via PAL and ICS is unclear. Additionally, free SA (FSA) and total SA (TSA) constitute two types of SA, and their respective associations with secondary metabolites under stress or exogenous SA treatment have not been fully elucidated in the majority of medicinal plants. *Scutellaria baicalensis* Georgi (Lamiaceae), or Baikal skullcap, is an herb that is used in traditional Chinese medicine. Baicalin and baicalein (Fig 1) are two well-known flavones isolated from the roots of *S*. *baicalensis* that have previously been shown to be the major bioactive flavones responsible for the efficacious properties of the plant in the treatment of fever, inflammation and cancer [13–15]. In this study, we subjected *S*. *baicalensis* seedlings to drought, salt stress, and exogenous SA treatment in a laboratory setting and tested the expression of *PAL* and *ICS*. The FSA, TSA, baicalin, and baicalein contents were determined, and the association between SA and PAL and ICS, and between FSA/TSA and baicalin and baicalein accumulation, respectively, were assessed in an attempt to elucidate the associated physiological response of the roots to drought stress, salt stress, and exogenous SA.

## Materials and methods

### Seedling culture and treatment conditions

Seedlings of *S*. *baicalensis* were grown from seed (purchased from the Medicinal Plant Cultivation Base of An Guo, China) and cultured according to the method of Su et al. [16]. For the stress treatments, 15% polyethylene glycol (PEG) 6000 was added to the soil for 3 d to mimic drought stress, and 150 mM NaCl was applied for 3 d to simulate salt stress. For the exogenous SA treatment, 70, 140, and 280 μM SA were respectively sprayed on the seedlings 24, 48 and 72h before harvesting. The roots of the seedlings were harvested, rinsed with ultrapure water, immediately frozen in liquid nitrogen, and stored at −80°C until further use.

### Total RNA extraction and reverse transcription

RNA was extracted from 0.01 g of root material. The root samples were homogenized with 400 μL extraction buffer (4 M guanidine isothiocyanate, 20 mM MES, 20 mM pH 7.0 EDTA, 50 mM β-mercaptoethanol) and the homogenate was centrifuged at 13,362 g at 4°C for 5 min. The supernatant was then transferred to a new tube and mixed with 500 μL chloroform-isopentanol and vortexed for 1 min. The mixture was centrifuged at 13,362 g and 4°C for 15 min, and the supernatant was transferred to a new tube and combined with 100 μL 3 M sodium acetate buffer and 350 μL ethanol. The mixture was maintained at −20°C for 30 min and then centrifuged at 13,362 g at 4°C for 20 min. The precipitate was re-suspended with 100 μL 3 M sodium acetate buffer and the suspension was centrifuged at 13,362 g and 4°C for 15 min. The precipitate was then washed with 800 μL 70% ethanol, dried at room temperature for 5 min, and then the dried precipitate was dissolved in 50 μL DEPC water.

In order to reduce DNA contamination, total RNA was treated with the DNA-Be-Gone-A kit (kit no. B641312, Sangon Biotech Co., Ltd. Shanghai, China) according to the kit manual, and the RNA quantity and quality were determined using a NanoDrop 2000 (Thermo Fisher Scientific Inc., Wilmington, USA).

Forty nanograms of total RNA was mixed with 1 μL of 10 μM oligo dT primer and 1 μL of 10 mM dNTPs, and diluted with RNA-free water to 12 μL. The mixture was kept at 65°C for 5 min and then rapidly cooled by placing on ice. Then, 4.8 μL reverse buffer, 1 μL RTase, and 0.6 μL RNase inhibitor were added to the mixture, and RNA-free water was added to yield a final volume of 24 μL. The mixture was kept at 42°C for 45 min, and then 95°C for 5 min.

### Cloning full length of *ICS* cDNA

The full length *ICS* cDNA was cloned according to the partial *ICS* sequence in *S*. *baicalensis* [16] using RACE. 3’- and 5’-RACE was conducted using the primer sequences listed in Table 1. The annealing temperature was set to 68°C. 5’-RACE was conducted with a kit according to the manual (Invitrogen, kit no. 18374–058).

**Table 1:** Primers for RACE PCR.

Name	Sequence (5’ to 3’)	Application
adaptor	GCTGTCAACGATACGCTACGTA- ACGGCATGACAGTGTTTTTTTTTTTTTTTTTT	adaptor
INTER-F	CTCAGGTTGAGTTTGATGAGCT	Inter-sequence amplification
INTER-R	CTCTGGAGTGTTTCCAATGAA	
GSP2-F	GCATCATGGGACTCAGCTGTCAATCAA	3’-RACE
GSP2-R	TCCTCTTGAGTGGCTGATGAGCTTGC	
outer	GCTGTCAACGATACGCTACGTAAC	
inner	GCTACGTAACGGCATGACAGTG	
GSP1-R2	GATGAAACCTGCCACATTGTTGCCTC	5’-RACE
GSP1-R1	ATGATGCCTTGCTAGGAACATGAGTCTG	
GSP-RT1	GGAGTGTTTCCAATGAATGCTGGTGAT	
AUAP	GGCCACGCGTCGACTAGTAC	
AAP	GCCACGCGTCGACTAGTACGGGGGGGGGG	

### Determination of gene expression levels

The relative expression levels of the *PAL* and *ICS* genes were determined by Real-Time (q)PCR (IQ2, Biorad Co., Ltd.) using the SYBR kit (kit No. 208056, Qiagen co., Ltd.). The primers CGAGTAGTGATTGGGTGATA and AGCCCTTGTTGCTGTATG were designed for *PAL* according to the sequence available on GenBank (HM062775.1). The primers TTGAAGGAAGTTCGATGATG and ATGAGTCTGGTGAAGCATAA were designed for *ICS* according to the full-length *ICS* cDNA. The data were analyzed using the 2^−ΔΔCt^ method [17].

### Enzyme extraction and activity determination

PAL activity was determined using High Performance Liquid Chromatography (HPLC) according to the modified method of Kovacik and Klejdus [18]. The samples were homogenized in borate buffer (pH 8.5) on ice, and the homogenates were immediately centrifuged at 20,879 g and 4°C for 20 min. The reaction mixture consisting of 200 μL supernatant and 200 μL 20 mM L-phenylalanine was incubated at 40°C for 1 h, and then 10 μL 6 M HCl was added. Parallel controls with HCl added prior to incubation were analyzed to determine the endogenous trans-cinnamic acid (CA) content. The HPLC assay was performed on an Agilent 1100 system equipped with a UV detector (G1314, Agilent, Santa Clara, CA, USA) and a 5 μm, 15 cm × 4.6 mm SUPELCOSIL^™^ LC-ABZ column (Sigma-Aldrich Co., St. Louis, MO, USA). PAL activity was expressed as CA content. The flow rate and elution gradient were determined as documented in Kovacik and Klejdus [18]. ICS activity was determined using a modified fluorescence analysis based on the method of Young and Gibson [19]. For the estimation of ICS activity, the samples were homogenized in extraction buffer (100 mM Tris-HCl at pH 7.5, 10% glycerol [v/v], 1 mM DTT, 0.2 mM PMSF, 1 mM EDTA) on ice and centrifuged at 20,879 g and 4°C for 20 min. The supernatant was desalted on a Sephadex G-25 column (Pharmacia, PD-10). The reaction mixture consisting of 125 μL desalted enzyme preparation and 125 μL reaction buffer (containing 1 mM Ba-chorismate, Sigma-Aldrich) was incubated at 30°C in the dark for 30 min. The reaction was stopped by the addition of 62.5 μL MeOH-*sec*-BuOH (1:1, v/v). The 100-μL reaction solution was mixed with 1.4 mL sodium phosphate buffer (0.1 M, pH 7.0), and then fluorescence was measured at an excitation wavelength of 305 nm and an emission wavelength of 410 nm. The solution was then heated at 100°C for 15 min, cooled to room temperature, and the fluorescence measured again. Blanks were produced by adding MeOH-*sec*-BuOH prior to the incubation step. The ICS activity was expressed as the net increase in fluorescence.

### Determination of SA

SA content was determined with HPLC according to the method of Dewdney et al. [20]. Briefly, root tissue samples (0.5 g) were homogenized with 3 mL extraction solution (90% MeOH, 0.55 mM o-anisic acid), vortexed, sonicated for 20 min, and then centrifuged at 9,279 g and 4°C for 20 min. The pellet was re-extracted with 2 mL 90% MeOH. The supernatants were divided into two equal-volume portions (for total SA and free SA measurements) and vacuum dried. For total SA samples, 500 μL β-glucosidase (80 U mL^−1^ in 100 mM sodium acetate pH 5.2, Sigma-Aldrich) was added to each sample. The samples were sonicated for 5 min, vortexed, covered with foil, and incubated for 90 min at 37°C. For both the total and free SA samples, 2.5 mL 5% trichloroacetic acid was added, and the samples were vortexed, sonicated for 5 min, and centrifuged at 9,279 g for 15 min. The supernatant was extracted twice with 2.5 mL extraction solution (ethyl acetate: cyclopentane, 1: 1). The organic phases were vacuum dried. Just prior to loading the samples on the HPLC, each was re-suspended in 250 μL of 20% MeOH and sonicated for 5 min.

HPLC was performed on an Agilent 1100 system equipped with a fluorescence detector (G1321A, Agilent, Santa Clara, CA, USA) and a 5 μm, 15 cm × 4.6 mm SUPELCOSIL^™^ LC-ABZ Plus Column (Sigma-Aldrich Co., St. Louis, MO, USA) preceded by a LC-ABZ Plus guard column. The flow rate and elution gradient were as documented in Dewdney [20]. SA and o-anisic acid were quantified using the fluorescence detector programmed to 302 nm excitation and 412 nm emission wavelengths.

### Determination of baicalin and baicalein

Baicalin and baicalein content were determined with HPLC according to the method of Su et al. [16]. Samples were diluted twice for injection and baicalin and baicalein were extracted from 0.1 g tissue. The roots were ground into a fine powder in liquid nitrogen and extracted with 500 μL ethanol. The homogenate was vortexed for 2 min and centrifuged at 13,362 g and 4°C for 15 min, and the supernatant assayed by HPLC. The HPLC assay was performed on an Agilent 1100 system equipped with a UV detector (G1314, Agilent, Santa Clara, CA, USA) and a 5 μm, 15 cm × 4.6 mm SUPELCOSIL^™^ LC-ABZ Plus Column (Sigma-Aldrich Co., St. Louis, MO, USA). The wavelength was set to 270 nm, the column was maintained at 35°C, and the injection volume was 5 μL. The flow rate and elution gradient were as documented in Su et al. [16]. The column was eluted with 27% acetonitrile for 3 min prior to the next injection.

### Statistics

Results from representative experiments were expressed as mean ± SE of nine independent repeats. Data from the experiments were analyzed by *t*-tests for simple comparisons between each treatment and its control. The correlations were analyzed using Spearman’s analysis. The assumptions of the analysis of variance were considered to be statistically significant at *P* < 0.05. All analyses were conducted in SPSS version 17.0.

## Results

### Cloning of full-length *ICS* cDNA

**Fig 2:**
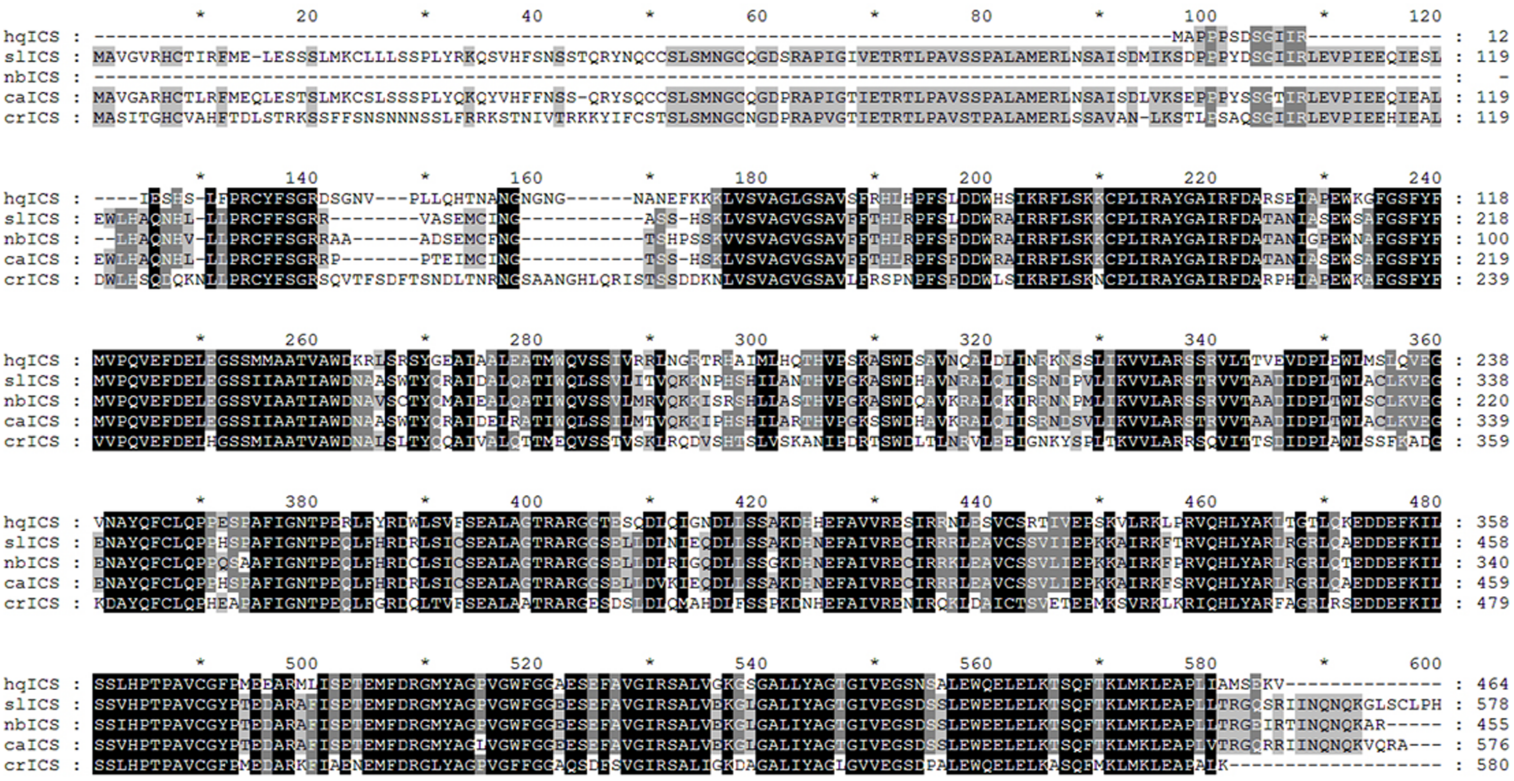
Alignment of the predicted protein sequence of *ICS* in *S*. *baicalensis* (the chorismate binding site is indicated in black.).

The full-length *ICS* cDNA in *S*. *baicalensis* was 1,801 bp and the coding sequence was 1,398 bp. Nucleotide blast indicated that the sequence had 76% similarity with its orthologs in *Solanum lycopersicum* (GenBank: NM_001247865.2), *Nicotiana benthamiana* (GenBank: EU257505.1), and *Capsicum annuum* (GenBank: NM_001325078.1), and 75% similarity with *Catharanthus roseus* (GenBank: AJ006065.1). Blastp based on the non-redundant protein database revealed that the predicted ICS protein in *S*. *baicalensis* had high amino acid sequence identity to its orthologs and possessed a conserved chorismate binding site (Fig 2) belonging to the super-gene family of chorismate binding proteins.

### Relative expression levels of *PAL* and *ICS*

**Fig 3:**
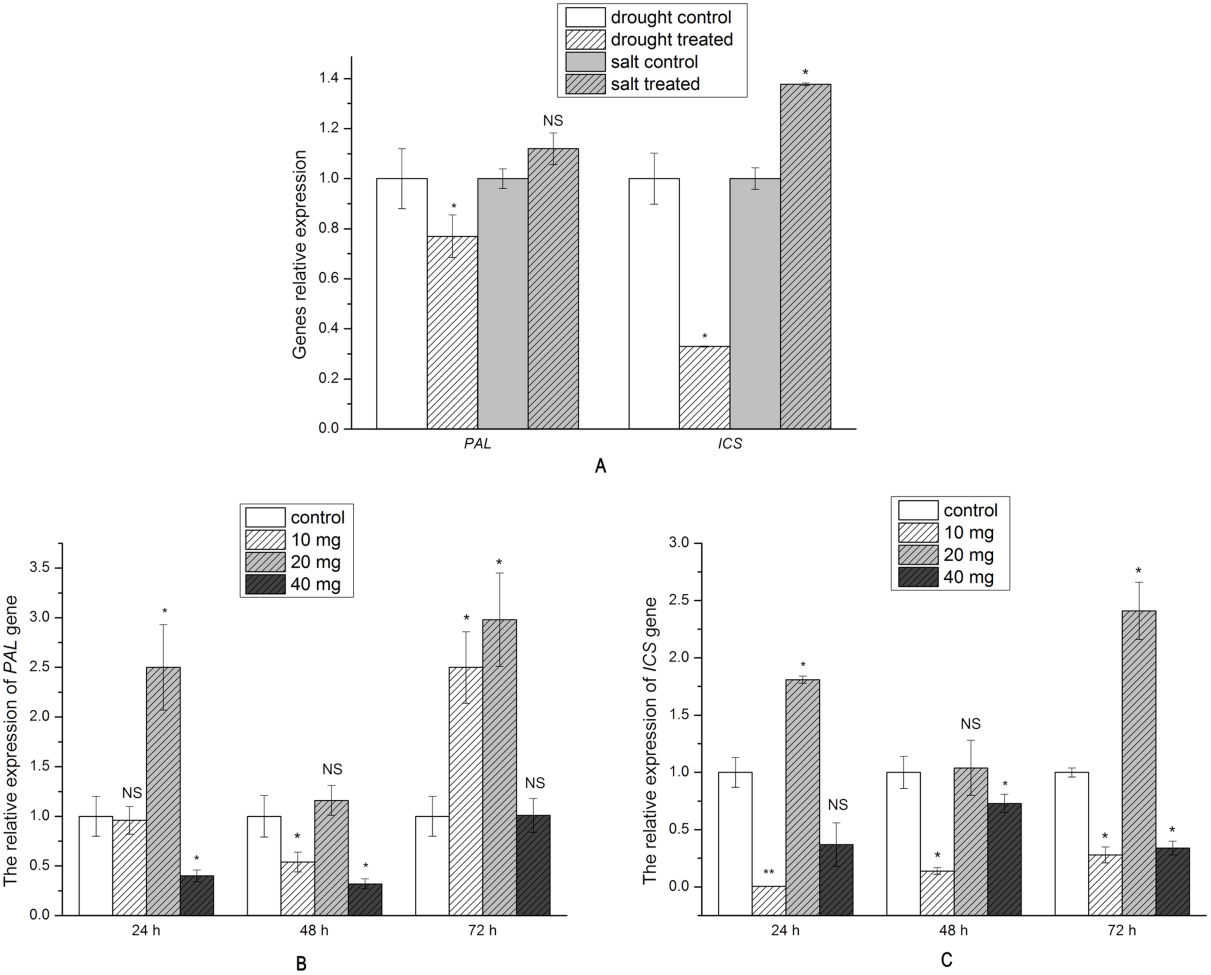
Relative expression of *PAL* and *ICS* in *S*. *baicalensis* roots (A) under drought and salt stress (B) and (C) in exogenous SA treatment (n = 9, * *P*<0.05, NS: non-significant).

*PAL* gene expression differed between the drought and salt stress treatments. The expression was down-regulated by 23% under drought stress, but up-regulated by 12% under salt stress; though this latter finding was not statistically significant (Fig 3A). Drought stress exhibited a more significant effect on *PAL* gene expression than salt stress. The expression of *ICS* was down-regulated significantly by 67% under drought stress, but significantly up-regulated by 38% under salt stress (Fig 3A).

Treatment with 140 μM SA resulted in the up-regulated expression of *PAL*, while the 70 and 280 μM treatments were associated with down-regulated expression at 24 and 48 h, followed by up-regulation at 72 h. The lowest *PAL* expression levels were detected at 48 h, where a 46% decrease was observed at 70 μM and a 68% decrease was observed at 280 μM. A non-significant expressional increase of 16% was detected in the 140 μM treatment. *PAL* gene expression peaked at 72 h, increasing 2.5-fold in the 70 μM treatment, 2.98-fold in the 140 μM treatment (Fig 3B).

*ICS* gene expression was up-regulated after 140 μM SA treatment, but was down-regulated following treatment with 70 or 280 μM SA. Treatment with 140 μM SA resulted in 1.81-, 1.04-, and 2.41-fold increased expression at 24, 48, and 72 h, respectively. The lowest expression level in the 70 μM treatment was detected at 24 h and corresponded to a decrease of 99.4%, while the lowest level in the 280 μM was observed at 72 h and corresponded to a 66% decrease. A non-significant 1.04-fold increase at 48 h constituted the lowest expression level in the 140 μM treatment (Fig 3C).

### PAL and ICS activity

**Fig 4:**
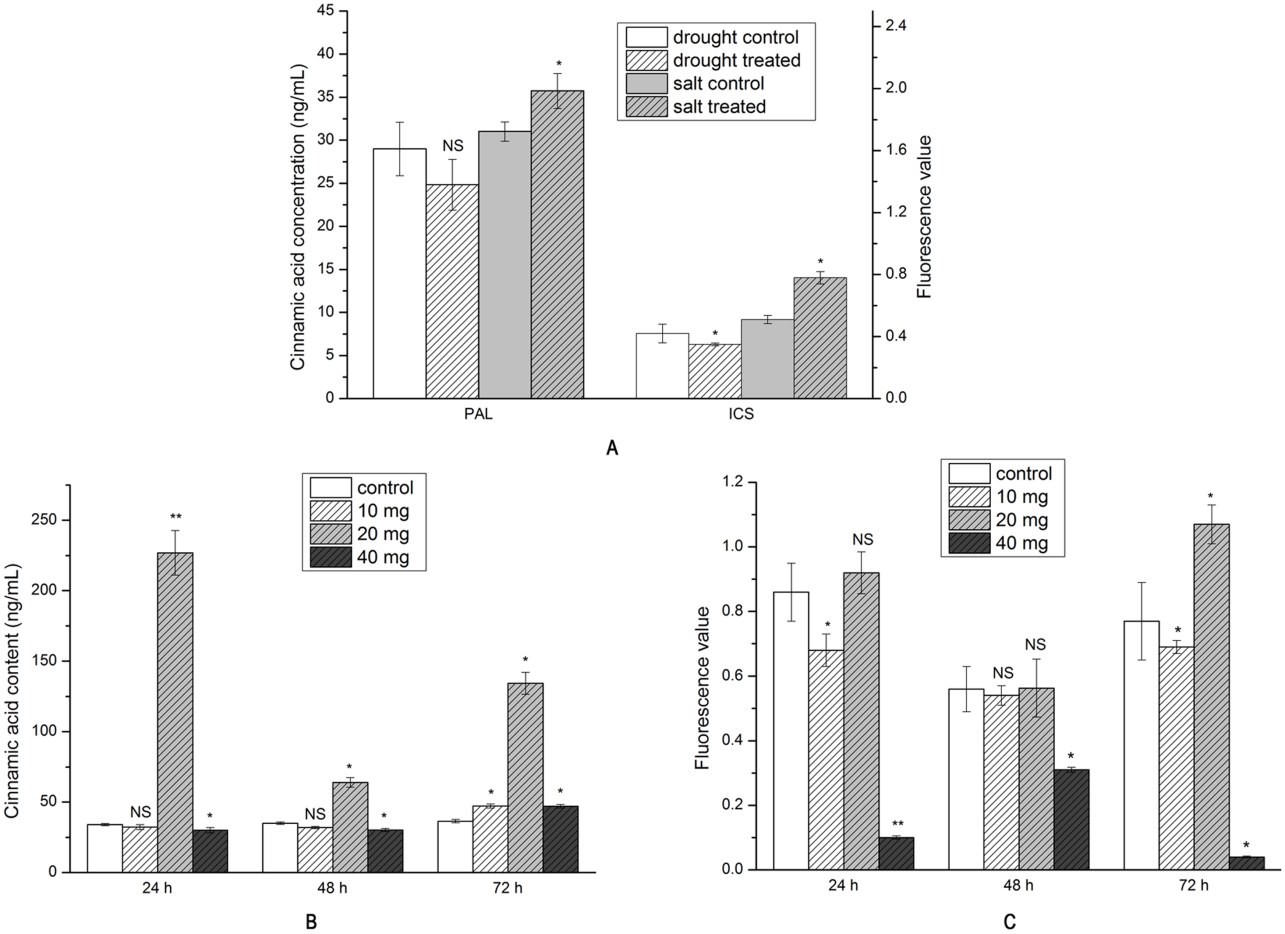
PAL and ICS activity in *S*. *baicalensis* roots (A) under drought and salt stress (B) and (C) in exogenous SA treatment (n = 9, * *P*<0.05, ** *P*<0.01, NS: non-significant).

PAL activity decreased by 14% under drought stress, though this was not statistically significant. Conversely, PAL activity increased significantly by 15% under salt stress (Fig 4A). ICS activity decreased significantly under drought stress (Fig 4A), while a significant increase of 53% was observed under salt stress (Fig 4A).

PAL activity was consistent with the gene expression data. Increased activity was observed after 140 μM SA treatment, with 6.67-, 1.83-, and 3.69-fold increases noted at 24 h, 48 h, and 72 h. As noted in the gene expression data, PAL activity decreased after 24 h and 48 h in the 70 μM and 280 μM treatments. The lowest activity was detected at 48 h, and corresponded to an 8.86% decrease at 70 μM and a 13.71% decrease at 280 μM, while the 1.83-fold increase observed in the 140 μM treatment was significant (Fig 4B).

ICS activity was broadly consistent with the gene expression patterns. Increased activity was associated with the 140 μM SA treatment, though the increase was only significant at 72 h. Conversely, the activity decreased after 70 μM or 280 μM treatment. The lowest activity in the 70 μM treatment was detected at 48 h and corresponded to a non-significant decrease of 3.57%, while a 94.81% decrease at 72 h was observed at 280 μM SA. A non-significant increase of 0.54% at 48 h was noted for the 140 μM treatment. Surprisingly, the ICS activity of the 70 μM treatment at 24 h did not corroborate the gene expression data (Fig 4C).

### SA content

**Fig 5:**
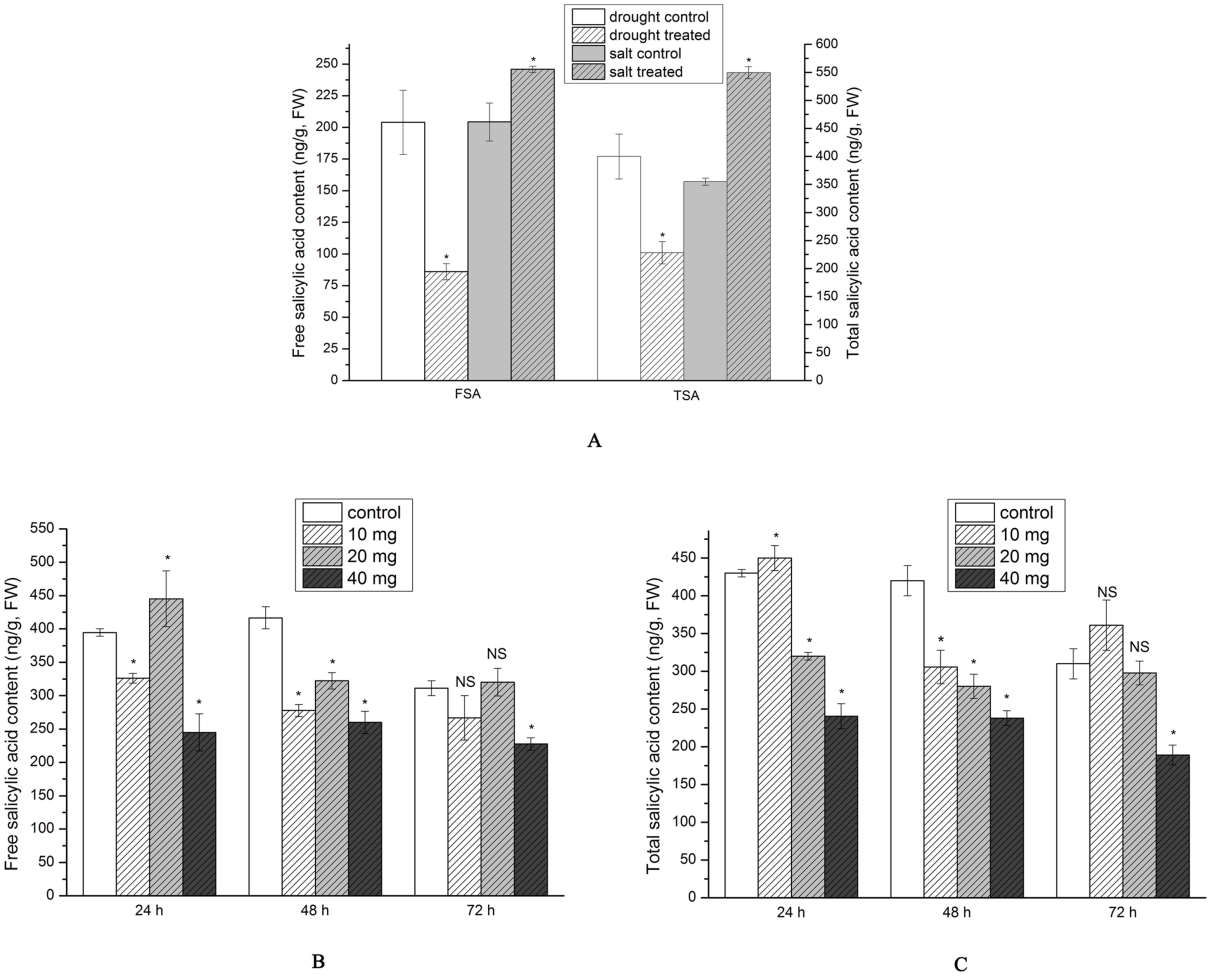
FSA and TSA content in *S*. *baicalensis* roots (A) under drought and salt stress (B) and (C) in exogenous SA treatment (n = 9, * *P*<0.05, NS: non-significant).

FSA decreased significantly to 86 ng·g^−1^ under drought stress, but increased significantly to 245.88 ng·g^−1^ under salt stress (Fig 5A). TSA content exhibited a similar pattern to FSA. The content decreased significantly by 43% to 228.23 ng·g^−1^ under drought stress, but increased significantly by 55% to 549.6 ng·g^−1^ under salt stress (Fig 5A).

FSA decreased in all the treatments, with the exception of a slight increase observed in the 140 μM treatment at 24 and 72 h. The lowest content in the 70 μM treatment was detected at 72 h and corresponded to a non-significant decrease of 14.28% to 266.67 ng/g, while a significant decrease of 26.78% to 227.78 ng/g was observed at 280 μM (Fig 5B).

TSA content decreased in most of the treatments, with the exception of the 70 μM treatment at 24 and 72 h; though this increase was non-significant. The lowest TSA content in the 70 μM treatment was detected at 48 h and corresponded to a 27.25% decrease to 305.56 ng/g. A decrease of 33.33% to 280 ng/g was observed at 48 h in the 140 μM treatment, while the lowest TSA content in the 280 μM treatment was noted at 72 h and corresponded to a 39.02% decrease to 189.03 ng/g (Fig 5C).

### Baicalin and baicalein content

**Fig 6:**
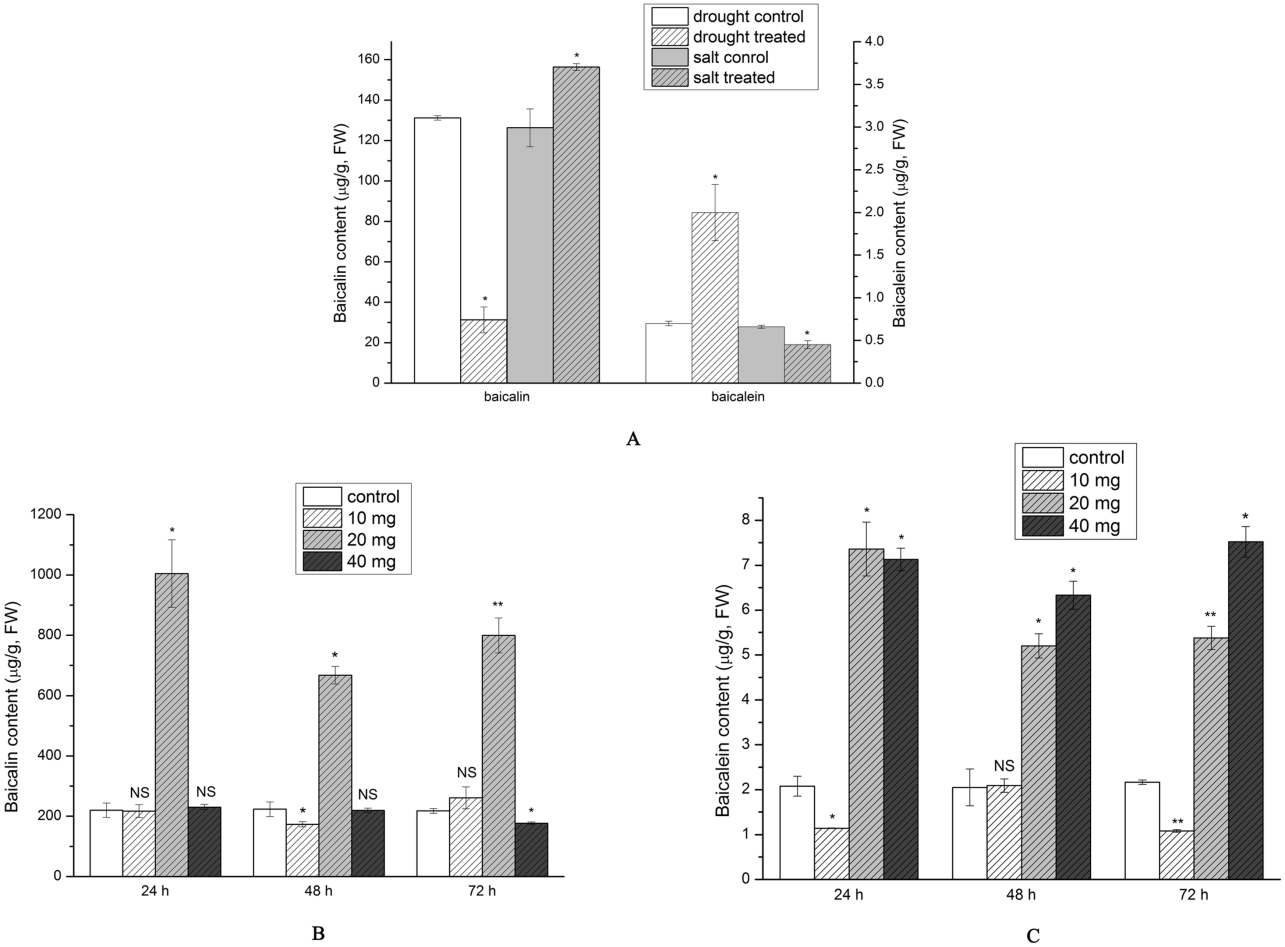
Baicalin and baicalein content in *S*. *baicalensis* roots (A) under drought and salt stress (B) and (C) in exogenous SA treatment (n = 9, * *P*<0.05, ** *P*<0.01, NS: non-significant.).

The baicalin content exhibited a sharp and significant decrease of 76.16% under drought stress to 31.28 μg·g^−1^. In contrast, under salt stress the content increased significantly by 23.77% to 156.36 μg·g^−1^ (Fig 6A). Conversely, baicalein content increased sharply by 185% under drought stress to 2 μg·g^−1^. A statistically significant decrease by 31.81% to 0.45 μg·g^−1^ was observed under salt stress (Fig 6A).

A sharp and significant increase in baicalin content was observed after 140 μM treatment, peaking at 1.01 mg/g at 24 h. A 20.05% increase to 261.31 μg/g baicalin occurred only at 72 h in the 70 μM treatment, while a 4.54% to 230.14 μg/g increase was observed at 24 h in the 280 μM treatment (Fig 6B).

Treatment with 140 and 280 μM SA significantly increased baicalein content, while the 70 μM treatment resulted in decreased baicalein content, with the exception of the 24 h time point. Baicalein content peaked at 24 h in the 140 μM treatment where it reached 7.36 μg/g, while a concentration of 7.52 μg/g was recorded at 72 h in the 280 μM treatment (Fig 6C).

### Correlation of SA with PAL, ICS, and metabolites

Under stress, both FSA and TSA were positively correlated with baicalin, though FSA exhibited a stronger and more significant correlation with baicalin in comparison to TSA. Both were significantly negatively correlated with baicalein (Table 2).

**Table 2:** Correlation of SA with PAL, ICS, and metabolites in the roots of *S*. *baicalensis* under stress.

	PAL	ICS	Baicalin	Baicalein
FSA	0.12	0.32	0.77*	−0.8*
TSA	0.14	0.43	0.5	−0.85*

*P< 0.05, n = 18

Following exogenous SA treatment, FSA was significantly correlated with PAL. They were also positively correlated with baicalin and negatively correlated with baicalein, and FSA exhibited a stronger and more significant correlation with baicalin in comparison to TSA (Table 3). The correlations of the exogenous SA treatment were broadly consistent with that of the stress treatment.

**Table 3:** Correlation of SA with PAL, ICS, and metabolites in the roots of *S*. *baicalensis* after exogenous SA treatment.

	PAL	ICS	Baicalin	Baicalein
FSA	0.883**	0.586	0.667*	-0.133
TSA	0.517	0.510	0.267	-0.65

** *P*< 0.01,

*P< 0.05,

n = 72

## Discussion

SA is synthesized via the PAL and ICS pathways in plants and the levels are closely associated with the activity of these two enzymes under stress. *PAL-* and *ICS*-silenced *S*. *baicalensis* seedlings were constructed in a previous study, and the effect of gene silencing on SA synthesis and the influence of endogenous SA on baicalin and baicalein accumulation were assessed. The study found that *PAL* silencing significantly influenced FSA content, while *ICS* affected the TSA content, and furthermore, FSA significantly influenced baicalin content, but not baicalein content [16]. However, these associations required further investigation, prompting us to investigate the correlation of PAL and ICS with SA, and the correlation of SA with baicalin and baicalein. Interestingly, the correlations were broadly consistent across both the stress and exogenous SA treatments. Both FSA and TSA exhibited a strong positive correlation with PAL and ICS, respectively, and FSA was significantly correlated with baicalin (Table 4).

**Table 4:** Correlation of SA with PAL, ICS, and metabolites in gene-silenced *S*. *baicalensis* roots.

	PAL	ICS	Baicalin	Baicalein
FSA	0.771	--	0.592*	−0.301
TSA	0.551	--	0.049	−0.219

*P<0.05,

-- enzyme activity not detected,

n = 12

Both free and bound SA were significantly reduced in whole plants of *A*. *thaliana* treated with the PAL inhibitor 2-aminoindan-2-phosphonic acid (AIP) under pathogenic fungal stress [21]. Four isoforms of *PAL* exist in the *Arabidopsis* genome, and SA levels were found to decrease sharply in quadruple mutants of *PAL* [22]. Although the inhibition of PAL or mutation of *PAL* isoforms results in a reduction in SA, considerable amounts of SA still accumulated under stress in *A*. *thaliana* [21, 22] and *Pueraria thomsonii* Benth. [23], implying that the ICS pathway is involved in SA synthesis. In comparison to the *PAL* quadruple mutant of *A*. *thaliana*, the *ICS1* mutant (*sid2*) accumulated far less total SA in response to pathogen infection [22]. The ICS pathway was found to be the major contributor to SA in *A*. *thaliana* under O_3_ exposure [24], and furthermore, was required for SA synthesis in plant defense [12]. Conversely, the PAL- and ICS-derived pathways contributed equally to pathogen-induced SA accumulation in soybean [25]. Our results indicated that both the PAL and ICS pathways are involved in SA synthesis in *S*. *baicalensis*, and SA was more strongly correlated with ICS than PAL under both drought and salt stress. Furthermore, drought stress resulted in a decrease in PAL and ICS activity accompanied by a decrease in SA, but under salt stress this was reversed, indicating that SA responds differently to different stresses.

As endogenous SA will be supplemented by the absorption of exogenous SA into the cell or organism, exogenous SA was applied to investigate its influence on the associated plant physiological processes. Interestingly, in a previous study, young pea seedlings grown from seed were soaked in ^3^H-labeled SA solution prior to sowing, and the results suggested that the increased endogenous SA observed in the seedlings, including free and bound SA, was synthesized *de novo* rather than taken up by the plant [26]. Our results indicated that FSA and TSA content decreased at most sampling time points after exogenous SA treatment, which was accompanied by fluctuations in PAL or ICS activity. Additionally, FSA was strongly positively correlated with PAL. Exogenous SA treatment can thus influence PAL activity. In another study, PAL activity was found to be dependent on exogenous SA content in *Taxus* cells, and increased following treatment with SA [27]. Additionally, the expression of *ICS* increased in the pea seedlings in which SA was *de novo* synthesized [26]. In combination, these results suggest that the fluctuations in PAL or ICS under exogenous SA treatment regulated the *de novo* synthesis of endogenous SA, as opposed to the absorption of exogenous SA.

Secondary metabolites play an important role in the interaction of plants with various stressors, and endogenous SA is intimately linked with the accumulation of plant secondary metabolites. Camalexin, an indolic secondary metabolite in *Arabidopsis*, was significantly reduced in *NahG* plants upon pathogen infection, in which the content of SA was reduced due to the expression of the *NahG* gene encoding bacterial salicylate hydroxylase [28]. The majority of phenylpropanoids were found to be negatively correlated with SA in transgenic *Populus*, in which endogenous SA levels were constitutively elevated [11]. Our results demonstrated that SA was positively correlated with baicalin, but negatively correlated with baicalein. The correlations observed between the exogenous SA treatment and the gene silencing results strengthened when the seedlings were subjected to drought and salt stress, which is expected given that most secondary metabolites function in improving stress resistance in plants. Furthermore, the regulation of secondary metabolites by SA is necessary for the efficient provision of intermediates in plants to maintain physiological homeostasis.

**Fig 7:**
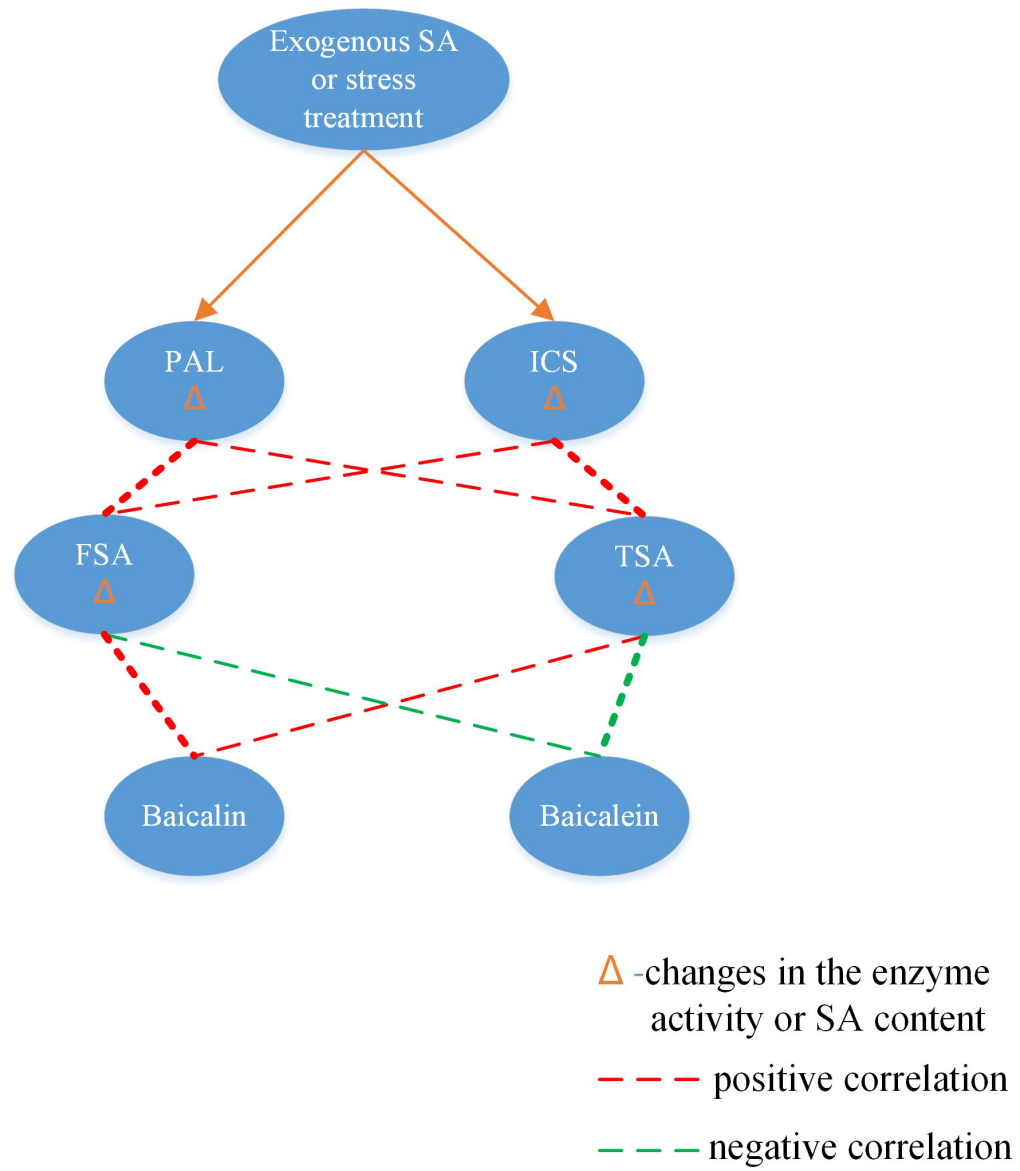
Correlation network in response to stress and exogenous SA treatment (Thick dashed lines indicates more high correlation.).

In conclusion, PAL and ICS function differently in the synthesis of FSA and TSA, FSA was strongly positively correlated with PAL. Both FSA and TSA positively correlated with baicalin, but negatively with baicalein (Fig 7). The findings of this study improve our understanding of the manner in which SA regulates secondary metabolites in *S*. *baicalensis*. In addition, the results of SA regulation in baicalin and baicalein production may be useful for exploring new strategies to improve the production in *S*. *baicalensis*. Thus, it provides an example of secondary metabolism regulation engineering in herbal plants.

## Supporting information

S1 Fig The amplification products of 3’RACE (A) and 5’RACE (B). (TIF)

S2 Fig The full length of *ICS* cDNA in *S*. *baicalensis* roots. (TIF)

S3 Fig HPLC chromatogram of baicalin and baicalein in *S*. *baicalensis* roots under drought and salt stress. (i) Standard chromatogram, peak 1 is baicalin, peak 2 is baicalein. (ii) control, (iii) drought stress, (iv) salt stress.
(TIF)

S4 Fig HPLC chromatogram of baicalin and baicalein in *S*. *baicalensis* roots in exogenous SA treatment. A-24 h, B-48 h, C-72 h; (i) Standard chromatogram, peak 1 is baicalin, peak 2 is baicalein. (ii) control, (iii) 70 μM SA, (iv) 140 μM SA, (v) 280 μM SA.
(TIF)

S1 Table Cinnamic acid concentration in PAL activity analysis (S1-1) under stress and (S1-2) in exogenous SA treatment. (DOCX)

S2 Table Fluorescence value in ICS activity analysis (S2-1) under stress and (S2-2) in exogenous SA treatment. (DOCX)

S3 Table FSA content in *S*. *baicalensis* roots (S3-1) under stress and (S3-2) in exogenous SA treatment. (DOCX)

S4 Table TSA content in *S*. *baicalensis* roots (S4-1) under stress and (S4-2) in exogenous SA treatment. (DOCX)

S5 Table Baicalin content in *S*. *baicalensis* roots (S5-1) under stress and (S5-2) in exogenous SA treatment. (DOCX)

S6 Table Baicalein content in *S*. *baicalensis* roots (S6-1) under stress and (S6-2) in exogenous SA treatment. (DOCX)
