# Supplementary figures and images for: Endogenous salicylic acid shows different correlation with baicalin and baicalein in the medicinal plant Scutellaria baicalensis Georgi subjected to stress and exogenous salicylic acid
Source: PLoS One. 2018 Feb 13;13(2):e0192114. doi: 10.1371/journal.pone.0192114 (PMC5810995; doi:10.1371/journal.pone.0192114)

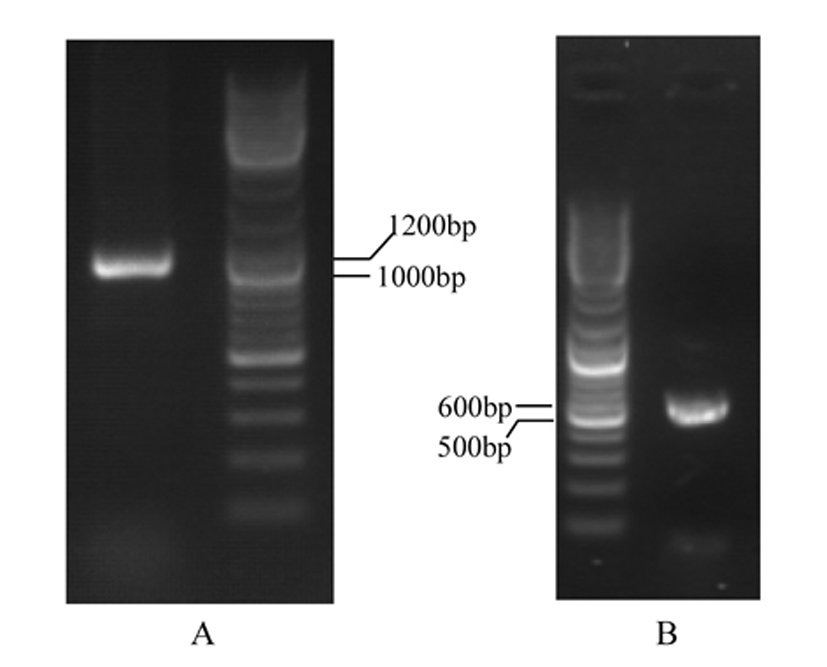

Supplement: S1 Fig — (TIF) [file pone.0192114.s001.tif]

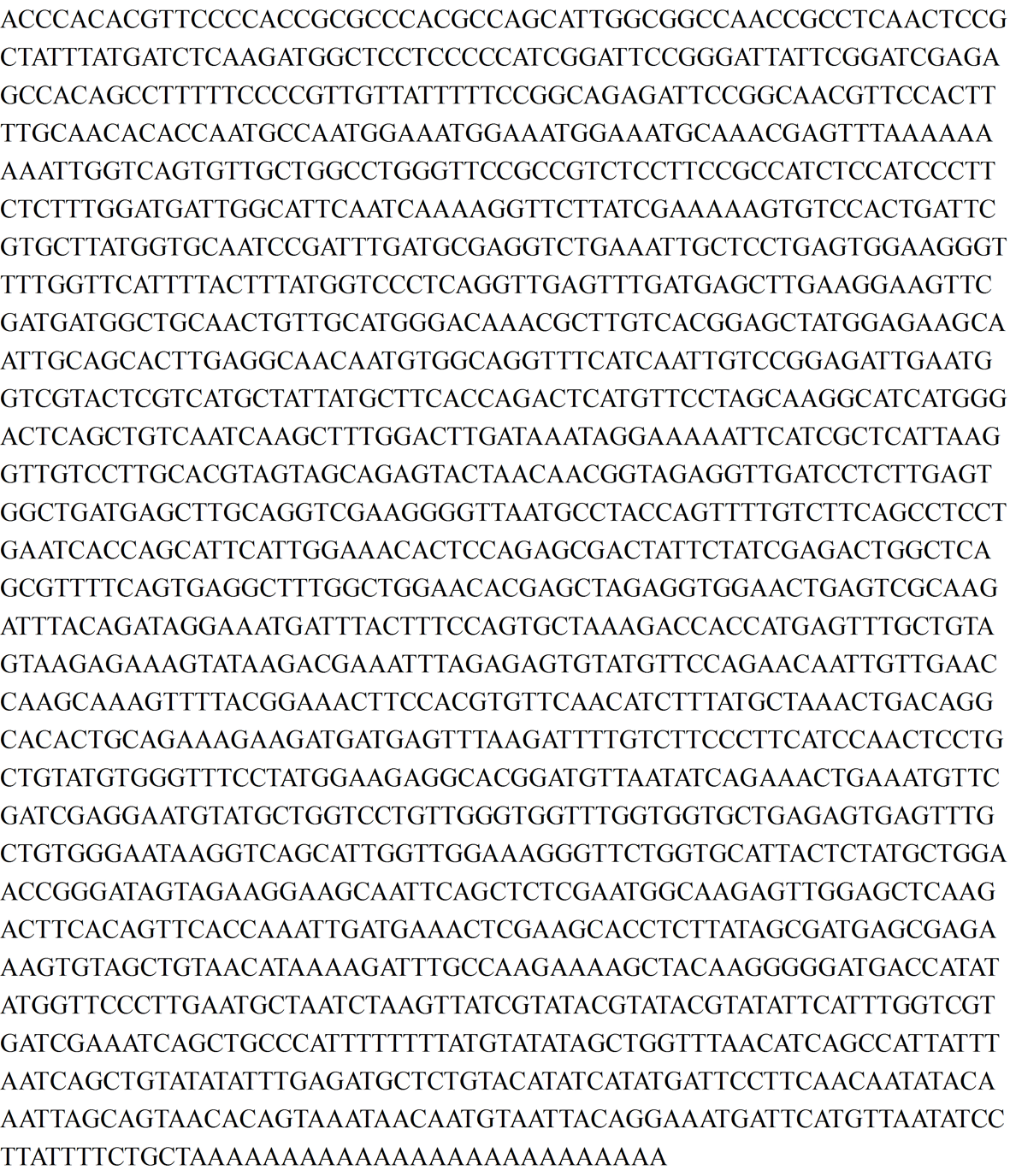

Supplement: S2 Fig — (TIF) [file pone.0192114.s002.tif]

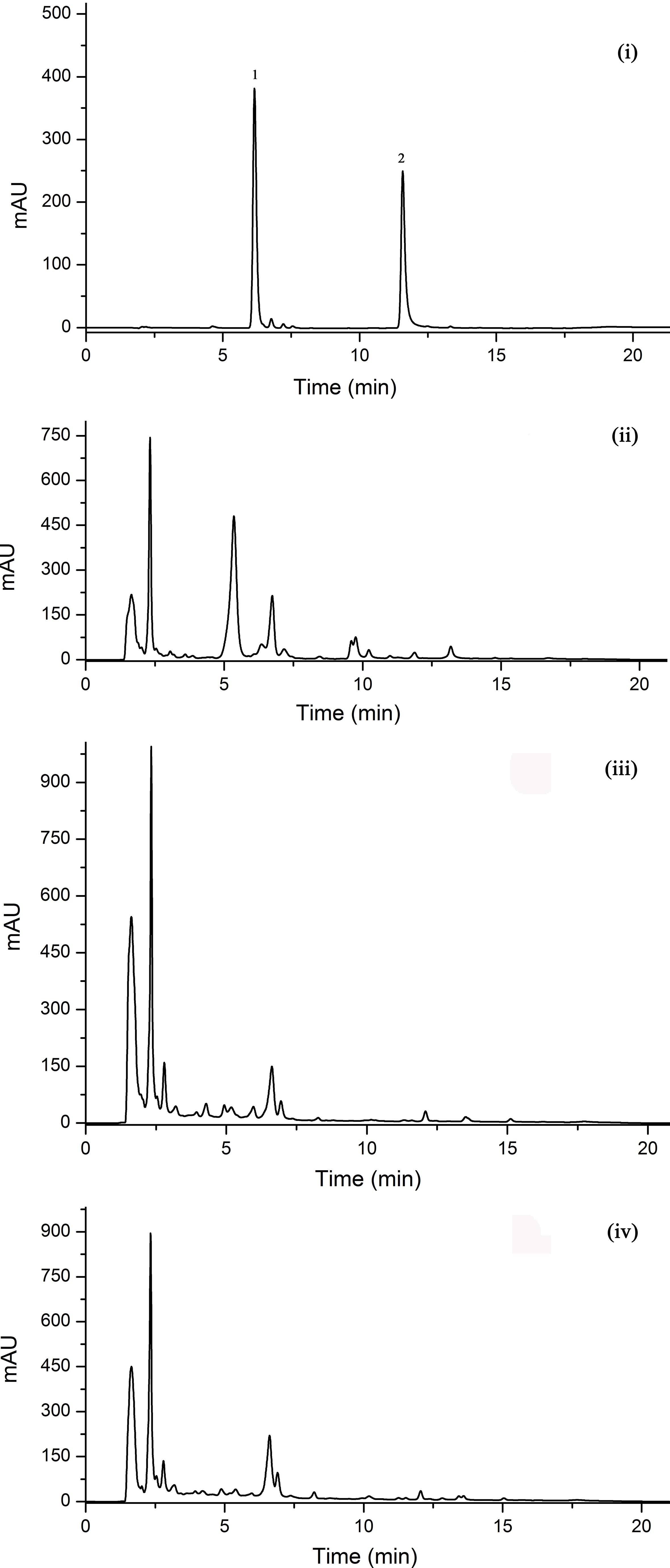

Supplement: S3 Fig — (i) Standard chromatogram, peak 1 is baicalin, peak 2 is baicalein. (ii) control, (iii) drought stress, (iv) salt stress. (TIF) [file pone.0192114.s003.tif]

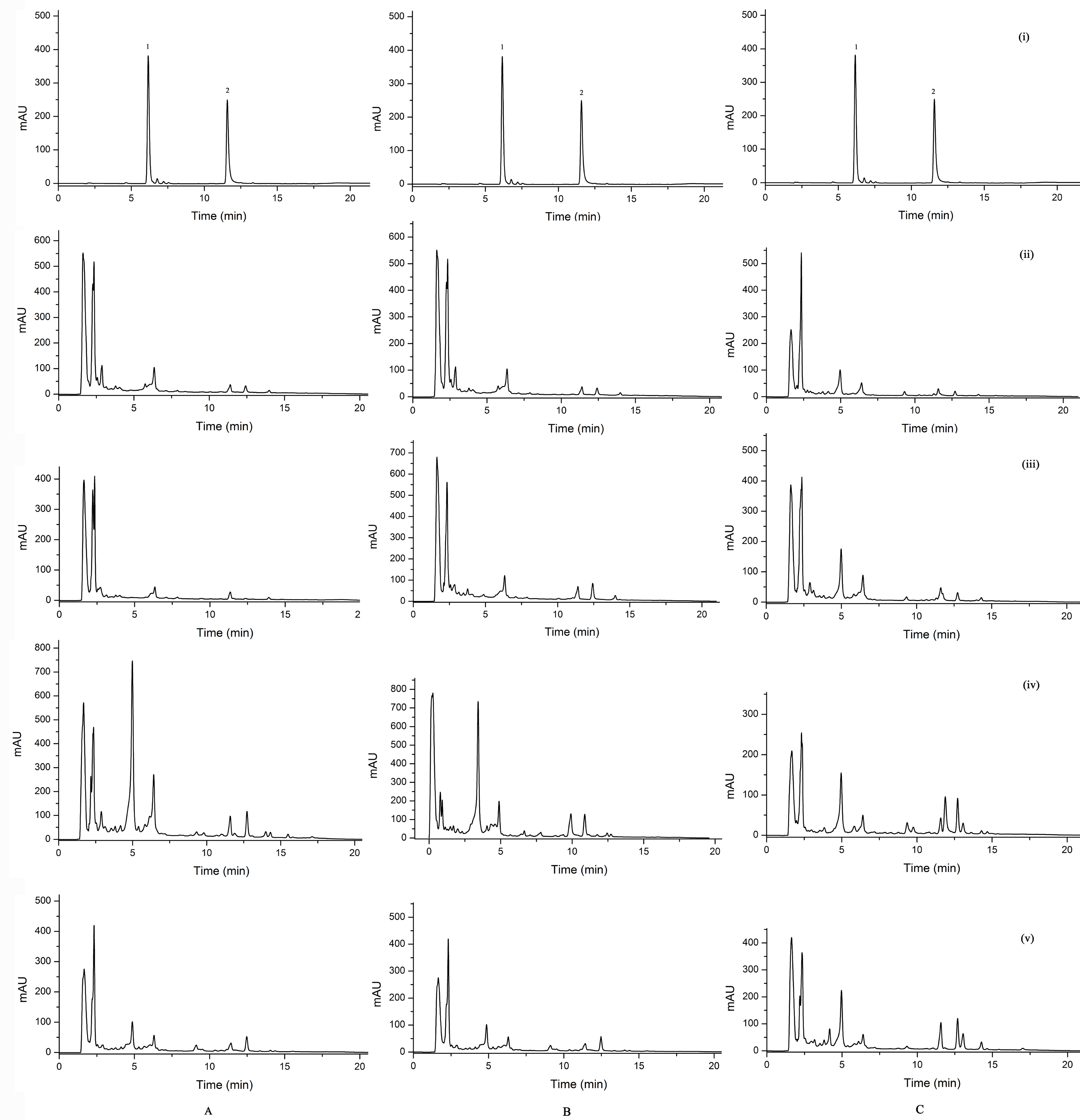

Supplement: S4 Fig — A-24 h, B-48 h, C-72 h; (i) Standard chromatogram, peak 1 is baicalin, peak 2 is baicalein. (ii) control, (iii) 70 μM SA, (iv) 140 μM SA, (v) 280 μM SA. (TIF) [file pone.0192114.s004.tif]
