# Supplementary material for: Endogenous salicylic acid shows different correlation with baicalin and baicalein in the medicinal plant Scutellaria baicalensis Georgi subjected to stress and exogenous salicylic acid
Source: PLoS One. 2018 Feb 13;13(2):e0192114. doi: 10.1371/journal.pone.0192114 (PMC5810995; doi:10.1371/journal.pone.0192114)
Supplement: S1 Table — (DOCX) [file pone.0192114.s005.docx]

S1 Table Cinnamic acid concentration in PAL activity analysis

S1-1 Table Stress treatment

| Stress condition | Control (ng/mL) | | Treated (ng/mL) |
| --- | --- | --- | --- |
| Drought | 29±3.07 | 24.83±2.95 | |
| Salt | 31.02±1.12 | 35.72±1.61* | |

* P<0.05.

S1-2 Table Exogenous SA treatment

| SA concentration (mg/L) | Time (h) | Control (ng/mL) | Treated (ng/mL) |
| --- | --- | --- | --- |
| 10 | 24 | 34±0.76 | 32.29±1.7 |
|  | 48 | 35±0.89 | 31.9±0.85 |
|  | 72 | 36.4±1.23 | 47.16±1.4* |
| 20 | 24 | 34±0.76 | 226.85±15.8** |
|  | 48 | 35±0.89 | 63.94±3.4* |
|  | 72 | 36.4±1.23 | 134.33±7.8* |
| 40 | 24 | 34±0.76 | 30±1.87* |
|  | 48 | 35±0.89 | 30.2±1.02* |
|  | 72 | 36.4±1.23 | 47.04±1.23* |

* P<0.05, **P<0.01.
