# Supplementary material for: Endogenous salicylic acid shows different correlation with baicalin and baicalein in the medicinal plant Scutellaria baicalensis Georgi subjected to stress and exogenous salicylic acid
Source: PLoS One. 2018 Feb 13;13(2):e0192114. doi: 10.1371/journal.pone.0192114 (PMC5810995; doi:10.1371/journal.pone.0192114)
Supplement: S2 Table — (DOCX) [file pone.0192114.s006.docx]

S2 Table Fluorescence value in ICS activity analysis

S2-1 Table Stress treatment

| Stress condition | Control | Treated |
| --- | --- | --- |
| Drought | 0.42±0.06 | 0.36±0.01* |
| Salt | 0.51±0.03 | 0.78±0.02* |

* P<0.05.

S2-2 Table Exogenous SA treatment

| SA concentration (mg/L) | Time (h) | Control | Treated |
| --- | --- | --- | --- |
| 10 | 24 | 0.86±0.09 | 0.68±0.05* |
|  | 48 | 0.56±0.07 | 0.54±0.03 |
|  | 72 | 0.77±0.12 | 0.69±0.02* |
| 20 | 24 | 0.86±0.09 | 0.92±0.07 |
|  | 48 | 0.56±0.07 | 0.563±0.09 |
|  | 72 | 0.77±0.12 | 1.07±0.06* |
| 40 | 24 | 0.86±0.09 | 0.1±0.01** |
|  | 48 | 0.56±0.07 | 0.31±0.01* |
|  | 72 | 0.77±0.12 | 0.04±0.003* |

* P<0.05, **P<0.01.
