# Supplementary material for: Endogenous salicylic acid shows different correlation with baicalin and baicalein in the medicinal plant Scutellaria baicalensis Georgi subjected to stress and exogenous salicylic acid
Source: PLoS One. 2018 Feb 13;13(2):e0192114. doi: 10.1371/journal.pone.0192114 (PMC5810995; doi:10.1371/journal.pone.0192114)
Supplement: S3 Table — (DOCX) [file pone.0192114.s007.docx]

S3Table FSA content in *S. baicalensis roots* (S3-1) under stress and (S3-2) in exogenous SA treatment.

S3-1 Table

| Stress condition | Control (ng/g) | Treated (ng/g) |
| --- | --- | --- |
| Drought | 203.99±25.42 | 86.06±6.4* |
| Salt | 204.27±15.02 | 245.88±1.97* |

* P<0.05.

S3-2 Table

| SA concentration (mg/L) | Time (h) | Control (ng/g) | Treated (ng/g) |
| --- | --- | --- | --- |
| 10 | 24 | 394.44±5.56 | 326.11±7.28* |
|  | 48 | 416.67±16.67 | 277.78±9.11* |
|  | 72 | 311.11±11.11 | 266.67±33.33 |
| 20 | 24 | 394.44±5.56 | 445.26±41.67* |
|  | 48 | 416.67±16.67 | 322.22±12.24 |
|  | 72 | 311.11±1.11 | 320.27±20.67 |
| 40 | 24 | 394.44±5.56 | 243.91±27.78* |
|  | 48 | 416.67±16.67 | 260±16.67* |
|  | 72 | 311.11±11.11 | 227.78±9.32* |

* P<0.05.
