# Supplementary material for: Endogenous salicylic acid shows different correlation with baicalin and baicalein in the medicinal plant Scutellaria baicalensis Georgi subjected to stress and exogenous salicylic acid
Source: PLoS One. 2018 Feb 13;13(2):e0192114. doi: 10.1371/journal.pone.0192114 (PMC5810995; doi:10.1371/journal.pone.0192114)
Supplement: S4 Table — (DOCX) [file pone.0192114.s008.docx]

S4 Table TSA content in S. baicalensis roots (S4-1) under stress and (S4-2) in exogenous SA treatment.

S4-1 Table

| Stress condition | Control (ng/g) | Treated (ng/g) |
| --- | --- | --- |
| Drought | 399.54±40.03 | 228.23±20.08* |
| Salt | 355.17±6.36 | 549.6±9.32* |

* P<0.05.

S4-2 Table

| SA concentration (mg/L) | Time (h) | Control (ng/g) | Treated (ng/g) |
| --- | --- | --- | --- |
| 10 | 24 | 430.07±5 | 450±16.67* |
|  | 48 | 420.01±20 | 305.56±22.22* |
|  | 72 | 310±20 | 361.11±33.34 |
| 20 | 24 | 430.07±5 | 320±5* |
|  | 48 | 420.01±20 | 280±16* |
|  | 72 | 310±20 | 297.67±15.81 |
| 40 | 24 | 430.07±5 | 240.56±16.67* |
|  | 48 | 420.01±20 | 238±9.82* |
|  | 72 | 310±20 | 189.03±13.22* |

* P<0.05.
