# Supplementary material for: Endogenous salicylic acid shows different correlation with baicalin and baicalein in the medicinal plant Scutellaria baicalensis Georgi subjected to stress and exogenous salicylic acid
Source: PLoS One. 2018 Feb 13;13(2):e0192114. doi: 10.1371/journal.pone.0192114 (PMC5810995; doi:10.1371/journal.pone.0192114)
Supplement: S5 Table — (DOCX) [file pone.0192114.s009.docx]

S5 Table Baicalin content in *S. baicalensis* roots (S5-1) under stress and (S5-2) in exogenous SA treatment.

S5-1 Table

| Stress condition | Control (µg/g) | Treated (µg/g) |
| --- | --- | --- |
| Drought | 131.28±1.09 | 31.28±6.17* |
| Salt | 126.33±9.4 | 156.36±1.46* |

* P<0.05.

S5-2 Table

| SA concentration (mg/L) | Time (h) | Control (µg/g) | Treated (µg/g) |
| --- | --- | --- | --- |
| 10 | 24 | 220.14±23.86 | 216.93±21.18* |
|  | 48 | 223.53±24 | 173.2±8.6* |
|  | 72 | 217.67±7.87 | 261.31±36.33 |
| 20 | 24 | 220.14±23.86 | 1010.67±112.1* |
|  | 48 | 223.53±24 | 267.6±29.45* |
|  | 72 | 217.67±7.87 | 799.33±58.21 |
| 40 | 24 | 220.14±23.86 | 230.14±9.12* |
|  | 48 | 223.53±24 | 219.41±7.11* |
|  | 72 | 217.67±7.87 | 176.66±4.09* |

* P<0.05.
