# Supplementary material for: Endogenous salicylic acid shows different correlation with baicalin and baicalein in the medicinal plant Scutellaria baicalensis Georgi subjected to stress and exogenous salicylic acid
Source: PLoS One. 2018 Feb 13;13(2):e0192114. doi: 10.1371/journal.pone.0192114 (PMC5810995; doi:10.1371/journal.pone.0192114)
Supplement: S6 Table — (DOCX) [file pone.0192114.s010.docx]

S6 Table Baicalein content in *S. baicalensis* roots (S6-1) under stress and (S6-2) in exogenous SA treatment.

S6-1 Table

| Stress condition | Control (µg/g) | Treated (µg/g) |
| --- | --- | --- |
| Drought | 0.7±0.03 | 2±0.33* |
| Salt | 0.66±0.04 | 0.45±0.05* |

* P<0.05.

S6-2 Table

| SA concentration (mg/L) | Time (h) | Control (µg/g) | Treated (µg/g) |
| --- | --- | --- | --- |
| 10 | 24 | 2.08±0.22 | 1.14±0.01* |
|  | 48 | 2.05±0.41 | 2.09±015* |
|  | 72 | 2.17±0.05 | 1.08±0.03 |
| 20 | 24 | 2.08±0.22 | 7.36±0.6* |
|  | 48 | 2.05±0.41 | 5.2±0.27* |
|  | 72 | 2.17±0.05 | 5.38±0.26 |
| 40 | 24 | 2.08±0.22 | 7.13±0.25* |
|  | 48 | 2.05±0.41 | 6.33±0.31* |
|  | 72 | 2.17±0.05 | 7.52±0.34* |

* P<0.05.
